# Potential Role for Peptidylarginine Deiminase 2 (PAD2) in Citrullination of Canine Mammary Epithelial Cell Histones

**DOI:** 10.1371/journal.pone.0011768

**Published:** 2010-07-26

**Authors:** Brian D. Cherrington, Eric Morency, Angela M. Struble, Scott A. Coonrod, Joseph J. Wakshlag

**Affiliations:** 1 J.A. Baker Institute for Animal Health, College of Veterinary Medicine, Cornell University, Ithaca, New York, United States of America; 2 Department of Clinical Sciences, College of Veterinary Medicine, Cornell University, Ithaca, New York, United States of America; University of Birmingham, United Kingdom

## Abstract

Peptidylarginine Deiminases (PADs) convert arginine residues on substrate proteins to citrulline. Previous reports have documented that PAD2 expression and activity varies across the estrous cycle in the rodent uterus and pituitary gland, however, the expression and function of PAD2 in mammary tissue has not been previously reported. To gain more insight into potential reproductive roles for PAD2, in this study we evaluated PAD2 expression and localization throughout the estrous cycle in canine mammary tissue and then identified possible PAD2 enzymatic targets. Immunohistochemical and immunofluorescence analysis found PAD2 expression is low in anestrus, limited to a distinct, yet sparse, subset of epithelial cells within ductal alveoli during estrus/early diestrus, and encompasses the entire epithelium of the mammary duct in late diestrus. At the subcellular level, PAD2 is expressed in the cytoplasm, and to a lesser extent, the nucleus of these epithelial cells. Surprisingly, stimulation of canine mammary tumor cells (CMT25) shows that EGF, but not estrogen or progesterone, upregulates PAD2 transcription and translation suggesting EGF regulation of PAD2 and possibly citrullination in vivo. To identify potential PAD2 targets, anti-pan citrulline western blots were performed and results showed that citrullination activity is limited to diestrus with histones appearing to represent major enzymatic targets. Use of site-specific anti-citrullinated histone antibodies found that the N-terminus of histone H3, but not H4, appears to be the primary target of PAD activity in mammary epithelium. This observation supports the hypothesis that PAD2 may play a regulatory role in the expression of lactation related genes via histone citrullination during diestrus.

## Introduction

The peptidylarginine deiminases (PADs) are a family of calcium-dependent enzymes that post-translationally convert arginine residues on substrate proteins to the non-standard amino acid citrulline. PAD catalyzed citrullination, with concomitant loss of the positive imine group, converts the strongly basic arginine residue to a neutral amino acid. Loss of basic charge caused by citrullination is thought to disrupt charge distribution within the substrate protein and alter its ability to interact with other molecules [1], [2]. The PAD family consists of five members (1–4 and 6) located within a gene cluster encompassing ∼300 kb at human chromosome 1p36.13. PADs 1 and 3 and PADs 4 and 6, respectively, are closely aligned [1], [3] while PAD2, the apparent ancestral homolog, is set apart from the other PADs on chromosome 1 and is oriented in the opposite direction. Additionally, PAD2 is the most widely expressed and largest of the PAD genes with a long, distinctive 3′ untranslated region (UTR). The PAD enzymes and citrullinated proteins are associated with multiple human diseases including rheumatoid arthritis, multiple sclerosis, Alzheimer's disease, and, more recently, with cancer [4]–[7].

PAD expression in mammary tissue has not been documented. However, previous reports have shown that PAD2 is expressed in other reproductive tissues in a hormone dependent manner. For example, both PAD2 and citrullination levels were found to be higher in the female rodent pituitary gland than in males and PAD2 was also found to be expressed in the luminal and glandular epithelia of the uterine endometrium with expression levels changing in an estrous cycle-dependent manner [8], [9]. Further, ovariectomized mice treated with estrogen (E_2_) displayed both increased PAD2 mRNA levels and increased citrullination in uterine samples compared to vehicle treated controls suggesting E_2_-mediated regulation.

Potential PAD2 targets in reproductive tissues have not been previously identified. However, two in vivo substrates for PAD2 have been described in other tissues: myelin basic protein (MBP) in neurons and vimentin in skeletal muscle and macrophages. In macrophages, the presence of high calcium levels cause PAD2 to citrullinate vimentin resulting in the breakdown of the vimentin intermediate filament network potentially to play a role in apoptotic events [10]. The brain expresses PAD2 where it citrullinates MBP a major component of the myelin sheath that covers the axons of nerves. MBP normally contains non-citrullinated arginine residues allowing compact myelin sheaths to form; citrullinated MBP is not capable of forming tight sheaths which is hypothesized to lead to neurodegeneration and possibly multiple sclerosis [5], [11]. There is also in vitro evidence that PAD2 can citrullinate arginine residues on histone H4 [12]. When incubated with histones, purified skeletal muscle PAD2 converts methyl arginine 3 histone H4 to citrulline, although no evidence yet exists to link PAD2 to histone citrullination in vivo.

A complete understanding of the role of PAD2 in vivo requires the utilization of animal models to follow PAD2 expression through hormonal fluctuations across the entire estrous cycle. Compared to mice, the dog mammary gland model is more closely in line with human breast tissue from a molecular, morphological, and hormonal prospective [13]. For example, during the estrous/menstrual cycle hormonal fluctuations in dog and human females show similar dramatic rises in E_2_ and progesterone (P_4_). Both female dogs and humans also show significant changes in mammary stromal tissue during the estrous cycle that does not occur in mice [14], [15]. Additionally, the dog is a suitable model for evaluation of estrous cycle and lactation-dependant mammary changes considering dogs facilitate full lactational capabilities regardless of parturition with each cycle. This difference may be due to a species' dependant persistent elevation of P_4_ throughout diestrus leading to a pseudopregnant state [14]. Therefore, here we have utilized canine mammary tissue to examine how and where PAD2 is expressed and the functional consequences of expression.

In this report, we show for the first time that PAD2 is expressed in the cytoplasm and nucleus of canine mammary alveolar luminal epithelial cells. Further, we show that PAD2 expression varies across the estrous cycle, with expression initiating during mammary gland proliferation in estrus/early diestrus and increasing through lactation during diestrus. Additionally, we show that PAD2 expression at diestrus directly correlates with citrullination of the histone H3 tail in mammary epithelial cells suggesting that PAD2 may play a novel role in gene regulation in the mammary gland.

## Materials and Methods

### Materials

Canine mammary tissue samples were collected from 6 dogs (2 in anestrus, 2 in estrus/early diestrus and 2 in late diestrus based on tissue morphologic changes) immediately after euthanasia in accordance with the guidelines outlined in the Report of the AVMA on Euthanasia under a Cornell University approved IACUC protocol (#2007-0073). Authors did not house the animals but collected tissues from intact female beagles between 3 and 5 years old which were being used for a different study as part of a collective effort to utilize all available tissues and decrease the number of animals used. Mammary tissue sections were snap frozen and stored at −80°C until utilized for protein and RNA experiments detailed below. At the same time, additional mammary tissue sections were placed in neutral buffered formalin and allowed to fix overnight. The following morning fixed mammary tissue was embedded, sectioned, and floated on micro-probe slides by the Cornell University Histology Core Laboratory. All canine mammary tissues were evaluated by a veterinary pathologist for stage of the estrus cycle, immunostaining and general morphology.

Canine mammary tumor 25 (CMT25) cells were acquired from the Cornell University comparative oncology program and grown in phenol red free RPMI 1640 (Invitrogen, Carlsbad, CA) supplemented with charcoal stripped 10% fetal bovine serum (FBS) (Invitrogen), 1% sodium pyruvate (Invitrogen), 1% HEPES buffer (Invitrogen), 1% sodium bicarbonate (Sigma-Aldrich, St.Louis, MO) and 1% antibiotic antimycotic (Sigma-Aldrich) [16]. For experiments, the cells were cultured in RPMI 1640 with 1% FBS for 24 hours and then treated with either 100 ng/ml of human epidermal growth factor (Invitrogen), 150 ng of sheep prolactin (Sigma-Aldrich) or 100 nM E_2_ (Sigma-Aldrich) or P_4_ (Sigma-Aldrich) for 24 hours and then harvested.

### Immunohistochemistry (IHC) and immunofluorescence (IF)

IHC and IF experiments were carried out using a standard protocol. Briefly, slides were rehydrated in 3X 5 minute washes in xylene followed by single sequential 5 minute washes in 100, 95, and 75% EtOH. Slides for IHC were then incubated for 10 minutes in 0.5% hydrogen peroxide in methanol to quench endogenous peroxidases, however this step was omitted for IF slides. Next, slides were submerged in 0.01 M sodium citrate and boiled 2X for 10 minutes to retrieve antigens. After cooling, slides were washed in 1X PBS and then blocked in 10% normal goat serum and 2X casein (Vector Labs, Burlingame, CA) for 20 minutes at room temperature in a humidified microprobe chamber. Next, slides were blotted to remove excess blocking solution and then primary antibody diluted in 1X PBS was added for 2 hours at room temperature. Antibody dilutions are as follows: anti-PAD2 (ProteinTech #122100-1-AP, Chicago, IL) 1∶100, anti-Cytokeratin (Dako MS3515, Carpinteria, CA) 1∶100, anti-p63 (NeoMarkers MS-1084-P, Fremont, CA) 1∶100, anti-Ki67 (Biogenex Labs BGX-Ki67, San Ramon, CA) 1∶50, biotinylated WGA (Vector Labs, B-1025), anti-pan-citrulline (Millipore 17-347, Billerica, MA) 1∶50. After washing three times, slides were incubated with a secondary antibody (biotinylated secondary for PAD2) diluted 1∶200 in 1X PBS for 1 hour at room temperature then washed 3X in PBS. IHC slides were incubated in DAB chromagen (Vector Labs) solutions according to the manufacturer's protocol, washed and then counterstained with hematoxylin and coverslip mounted. IF slides were incubated in avidin conjugated-FITC (Vector Labs), washed and then mounted using Vectashield containing DAPI (Vector Labs). IHC and IF for pan-citrulline require an additional step after antigen retrieval due to the necessity of chemical modification of citrulline residue for antibody recognition. Slides were incubated in acid solution overnight at 37°C to modify the citrulline residues according to the manufacturer's protocol (Millipore). The following morning, slides were rinsed in ddH_2_O 3X before proceeding to the blocking step described above. During each experiment, duplicate slides were treated with control rabbit IgG antibody at the appropriate concentrations as a negative control.

### Western blotting

Homogenized mammary tissue and CMT25 cells were lysed in RIPA buffer containing 20 mM Tris (pH 8.0), 137 mM NaCl, 10% glycerol, 1% NP-40, 0.1% SDS, 0.5% deoxycholate, and 0.2 mM PMSF and 1X general protease inhibitor. Protein concentration in lysates was determined by Bradford assay prior to gel loading to ensure equal protein loading. 6X sample buffer (300 mM Tris-HCl, pH 6.8, 60% glycerol, 30 mM DTT, 6% SDS) was added to yield a final concentration of 1X and lysates were boiled at 95°C for 5 min. Samples were subjected to SDS polyacrylamide gel electrophoresis on a 10 or 15% gel (acrylamide:bis-acrylamide ratio of 29∶1) and electro-blotted to Immobilin PVDF membranes (Millipore). Membranes were blocked in 1X casein diluted in Tris buffered saline (TBS). Anti-PAD2 (ProteinTech), H3cit 2-8-17 (Abcam ab77164, Cambridge, MA), H3cit 26 (Abcam ab19847), H4cit 3 (Millipore 07-596), and Histone 3 antibody (Upstate 05-499) were diluted 1∶1000 and incubated overnight at 4°C. Blots were washed and then incubated with a 1∶10,000 dilution of anti-rabbit or anti-mouse conjugated HRP (Jackson ImmunoResearch Labs, West Grove, PA) for 2 hr at room temperature. All blots were washed for 60 min (6×10 min) with TBS-Tween after secondary antibody and then visualized by chemiluminescence using Millipore Immobilon Western. To confirm equal protein loading, membranes were stripped and re-probed with anti-β-actin (Abcam ab8227) or anti-cytokeratin (Dako) diluted 1∶2000. Western blots for pan-citrulline were electrophoresed and transferred according to standard protocols, however prior to blocking, membranes were subject to an overnight acid treatment at 37°C to chemically modify the citrulline residues according to the manufacturer's protocol (Millipore). The following morning, membranes were washed in ddH_2_O three times and then blocked and probed with the pan-citrulline antibody (1∶1000). Densitometry measurements were obtained from three independent western blots and measured using the BioRad Versdoc 4000. All treatment groups are compared to vehicle treated control in each experiment.

### qPCR

Three independent samples of frozen canine mammary tissue from each of the three estrus cycle stages were cut in >0.5 cm sections and placed in RNA*later*-Ice (Ambion, Austin, TX) for 24 hours to help stabilize the mRNA. After 24 hours, tissue was transferred to a new tube containing Qiagen RLT lysis buffer and homogenized (Qiagen, Valencia, CA). Messenger RNA was purified according to the Qiagen RNeasy protocol including on column DNAse treatment to remove genomic DNA. The resulting mRNA purified from canine mammary tissue was quantified and 0.5 µgs reverse transcribed using ABI High Capacity RNA to cDNA kit according to manufacturer's protocol (ABI, Foster City, CA). Complementary DNA was subject to qPCR analysis with intron spanning primers specific for the 75 kDa isoform of PAD2 (FWD 5′-TGAGAGCCTCGTGCAAGAGAACC-3′ and REV 5′-CGTGAGCCCCAACTCCTTCTTG-3′). Ribosomal protein S5 (RPS5) is a validated canine reference gene and was used as our endogenous control for qPCR analysis (FWD 5′-TCACTGGTGAGAACCCCCT-3′ and REV 5′-CCTGATTCACACGGCGTAG-3′) [17]. Quantitative PCR data was analyzed by the delta/delta Ct method in which all PAD2 Ct values are adjusted to corresponding RPS5 levels. For estrus cycle stage and hormone/growth factor treatment comparisons, the anestrus or vehicle treated control samples are normalized to 1 and all values are expressed as the mean ± SEM.

### Statistical Analysis

All experiments were independently repeated at least three times. Values were expressed as the mean ± the SEM. Means were separated by one-way ANOVA using Tukey's HSD and P<0.05 was considered statistically significant.

## Results

### Canine PAD2 is expressed in the mammary gland and its expression varies in an estrous cycle-dependent manner

Canine mammary tissue undergoes pronounced morphological rearrangement over the course of the estrous cycle. Although hormonal changes clearly facilitate this dramatic reorganization, the downstream mechanisms by which these hormones carry out this transformation remain unclear.

To begin investigating the possibility that PAD2 may play a role in these reorganization events, we obtained mammary tissue from naturally cycling beagles. Mammary tissue sections were staged by a veterinary pathologist using morphological criteria as described in a recent review article [18] and were classified as being in one of three stages: anestrus (showing involution), estrus/early diestrus (showing edema, and stromal and epithelial proliferation) or late diestrus (showing active lactation).

**Figure 1:**
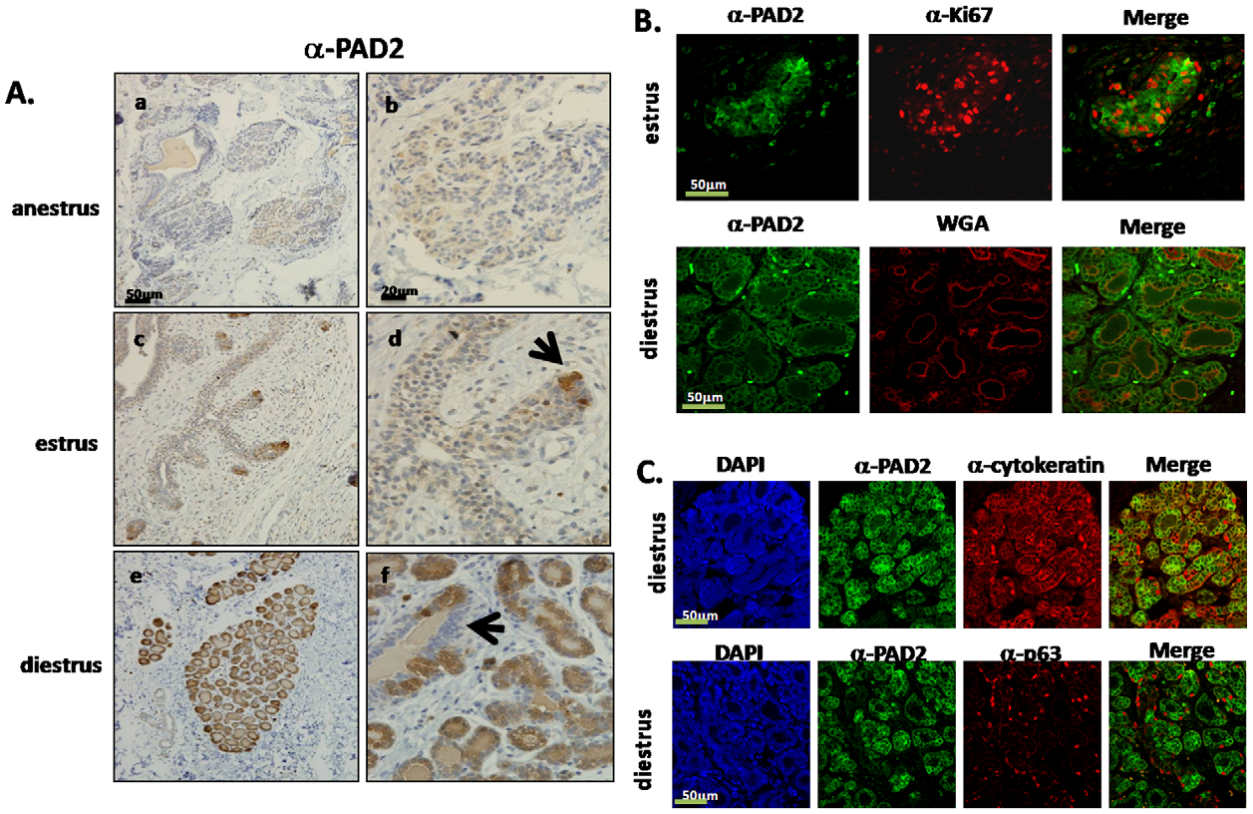
PAD2 expression in canine mammary tissue. (A) PAD2 is expressed in the canine mammary gland in an estrous cycle dependant fashion with the highest expression in alveolar unit epithelium during late diestrus. Anestrus (a 200×, b 630×), estrus/early diestrus (c 200×, d 630×), and late diestrus (e 200×, f 630×) mammary tissue sections were probed with anti-PAD2 antibody or equal concentration of rabbit IgG as a control (see Supplemental Figure 1A) and counterstained with hematoxylin. Arrow in estrus/early diestrus depicts occasional alveolar end units expressing PAD2. Arrow in diestrus depicts lack of PAD2 epithelial staining in ductule. (B) PAD2 (green fluorescent signal) is first detected in estrus/early diestrus, a time of high cellular proliferation, but PAD2 is widely expressed in late diestrus during active lactation. In estrus the red fluorescent signal represents the proliferation marker Ki67 (400×), while in diestrus the red fluorescent signal is WGA (400×) which stains the apical membrane of actively lactating mammary glands and all nuclei are stained with DAPI. (C) PAD2 expression is restricted to canine mammary luminal epithelial cells. PAD2 expression is shown as green fluorescent signal during anestrus and diestrus. In 3A, the red fluorescent signal represents cytokeratin, a marker for luminal epithelial cells, while in 3B the red signal is p63, a marker of myoepithelial cells, and all nuclei are stained with DAPI (400×).

Canine mammary tissue at anestrus displayed limited poorly differentiated nests of epithelium and this epithelium showed weak PAD2 staining (Figure 1A a–b). Interestingly, mammary tissue in estrus/early diestrus showed characteristic alveolar proliferation as confirmed with Ki67 staining (Figure 1B). PAD2 staining in this stage is sparse and primarily localized to the scattered expanding end terminal alveolar units (Figure 1A c–d, arrow). However, by diestrus when mammary ducts are well differentiated, PAD2 expression was present in all the alveolar units and this signal was not detected in either ductules or ducts (Figure 1A e–f, arrow). In stark contrast, PAD4 IHC staining of canine mammary tissue sections indicate that PAD4 staining is lowest during late diestrus supporting the hypothesis of a unique role for PAD2 during late diestrus (Figure S1B).

To confirm staging of late diestrus samples and milk production, we co-stained our tissues with wheat germ agglutin (WGA), which stains the apical membrane of actively lactating mammary glands. Results showed that PAD2 expression also strongly correlates with WGA staining at diestrus, thus potentially linking PAD2 expression with lactation (Figure 1B). Finally, we used TUNEL staining to determine if any of our estrous cycle staged mammary tissue samples show high levels of apoptosis as occurs during mammary gland involution. None of our samples showed a high degree of apoptosis/necrosis indicating that the tissues were not actively involuting; however the anestrus sections did display the most apoptotic cells (data not shown). Thus, by both morphological and cellular IF labeling, PAD2 expression varies over the course of the canine estrous cycle with highest expression levels appearing in alveolar unit epithelium during diestrus.

Since we demonstrate that PAD2 is expressed in mammary tissue, we next sought to define which cell types express PAD2 during the different phases of the canine estrous cycle. To accomplish this objective, we used IF microscopy on the same tissue sections utilized in the IHC experiments. To label and visualize specific cell populations within the mammary tissue we used antibodies specific for PAD2, cytokeratin (an epithelial marker), p63 (a myoepithelial marker) [19], or DAPI (nuclear stain). Regarding PAD2 tissue localization, results showed that, similar to cytokeratin, PAD2 is primarily expressed in epithelial cells. Co-staining with the anti-p63 antibody was then carried out to investigate if PAD2 primarily localized to myoepithelial or luminal epithelial cells. Results showed that PAD2 expression does not overlap with myoepithelial p63 positive cells and is thus mainly confined to mammary luminal epithelial cell populations (Figure 1C).

### PAD2 mRNA and protein levels are highest during canine diestrus

**Figure 2:**
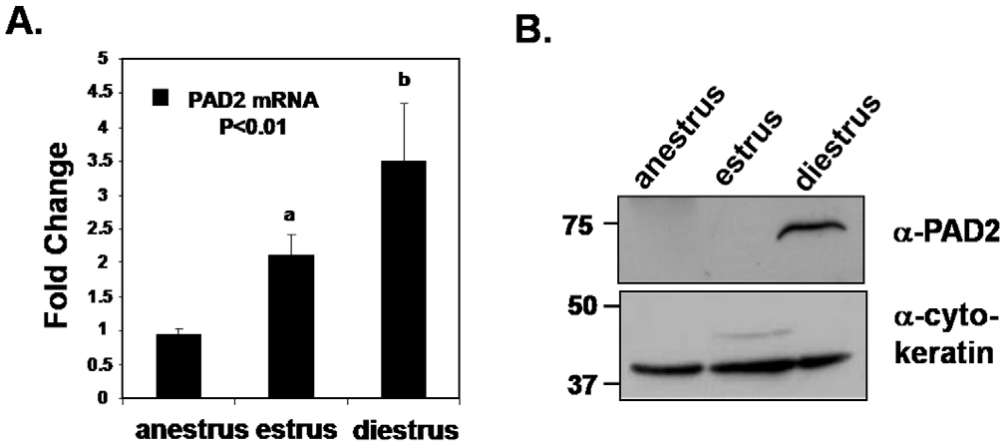
PAD2 mRNA and protein are differentially expressed across the canine estrous cycle with highest levels detected during late diestrus. (A) RNA was purified from canine mammary tissue samples, reverse transcribed and resulting cDNA was used in qPCR reactions with intron spanning primers specific for the 75 kDa isoform of PAD2 and RPS5, a characterized canine reference gene. All values are normalized to anestrus samples and bars represent the means ± SEM. Means were separated by one-way ANOVA using Tukey's HSD and the letters a and b represent significant differences (P<0.01). (B) Canine mammary tissue samples from anestrus, estrus/early diestrus and late diestrus were subject to SDS-PAGE and probed with an anti-PAD2 antibody which detected a 75 kDa band, the estimated molecular weight of PAD2.

We next examined PAD2 mRNA and protein levels from the same staged tissue extracts by qPCR and western blot in an effort to corroborate our IHC findings. Intron spanning primers specific to the catalytically active 75 kDa isoform of PAD2 were used to detect PAD2 mRNA and expression was normalized to a well characterized canine reference gene, RPS5 [17]. Messenger RNA levels were seen to significantly increase approximately 1 fold from anestrus to estrus with an additional fold increase being observed from estrus to diestrus (P<0.01) (Figure 2A). In concordance with our IHC and qPCR analysis, staged mammary tissue protein samples were examined by SDS-PAGE and confirmed that PAD2 expression is highest in late diestrus (Figure 2B). Additionally, the PAD2 antibody used in both the IHC and western blot experiments is specific for the 75 kDa isoform of PAD2 and does not appear to cross-react with other PAD family members (Figure S2).

### PAD2 expression is induced by EGF in the Canine Mammary Tumor (CMT) 25 primary mammary carcinoma epithelial cell line

**Figure 3:**
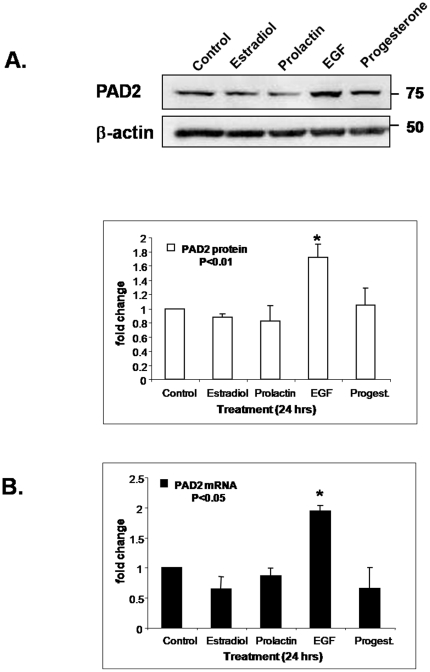
Treatment of the canine mammary tumor (CMT25) cell line with EGF results in an increase in PAD2 expression levels. (A) Western blot illustrating that only EGF treatment increases PAD2 protein levels with β-actin shown as a loading control. Densitometry measurements quantified treatment groups compared to vehicle treated control in three independent experiments and bars represent the means ± SEM. * indicates a statistically significant difference from other treatments (P<0.01) (B) Quantitative PCR analysis revealed elevated PAD2 transcript levels in CMT25 cells treated with EGF compared to vehicle treated control and * indicates a statistically difference from the other treatments (P<0.05).

Given the observed estrous cycle dependant changes in expression of PAD2 in canine mammary tissue samples, we decided to directly test the hypothesis that PAD2 expression is regulated by specific hormones and/or growth factors in mammary epithelial cells. Thus, we examined the effect of hormones and growth factors such as E_2,_ EGF, prolactin and P_4_ on PAD2 expression in canine mammary epithelial cells using two canine mammary tumor-derived cell lines: CMT12 and CMT25 cells [16], [20]. Preliminary experiments found that PAD2 was only expressed in the CMT25 cell line, indicating differential regulation of PAD2 expression across these two cell lines (data not shown). Our analysis of the CMT25 cells, however, found that this line constitutively expressed PAD2 in the presence of low serum levels. Given estrous cycle dependent changes in PAD2 expression, we predicted that E_2_, P_4_, or prolactin would potentially induce PAD2 expression. Surprisingly, however, treatment of CMT25 cells with these hormones did not significantly alter PAD2 expression with the exception of cells treated with EGF, where PAD2 expression was significantly increased (P<0.01) (Figure 3A). Further examination of PAD2 expression by qPCR of hormone/growth factor treated cells confirmed that PAD2 transcription was only affected by EGF stimulation (P<0.05) (Figure 3B). Presently, the specific signal transduction pathway responsible for EGF induced expression of PAD2 is under investigation.

### PAD2 is expressed in both the cytoplasmic and nuclear compartment of mammary epithelial cells

**Figure 4:**
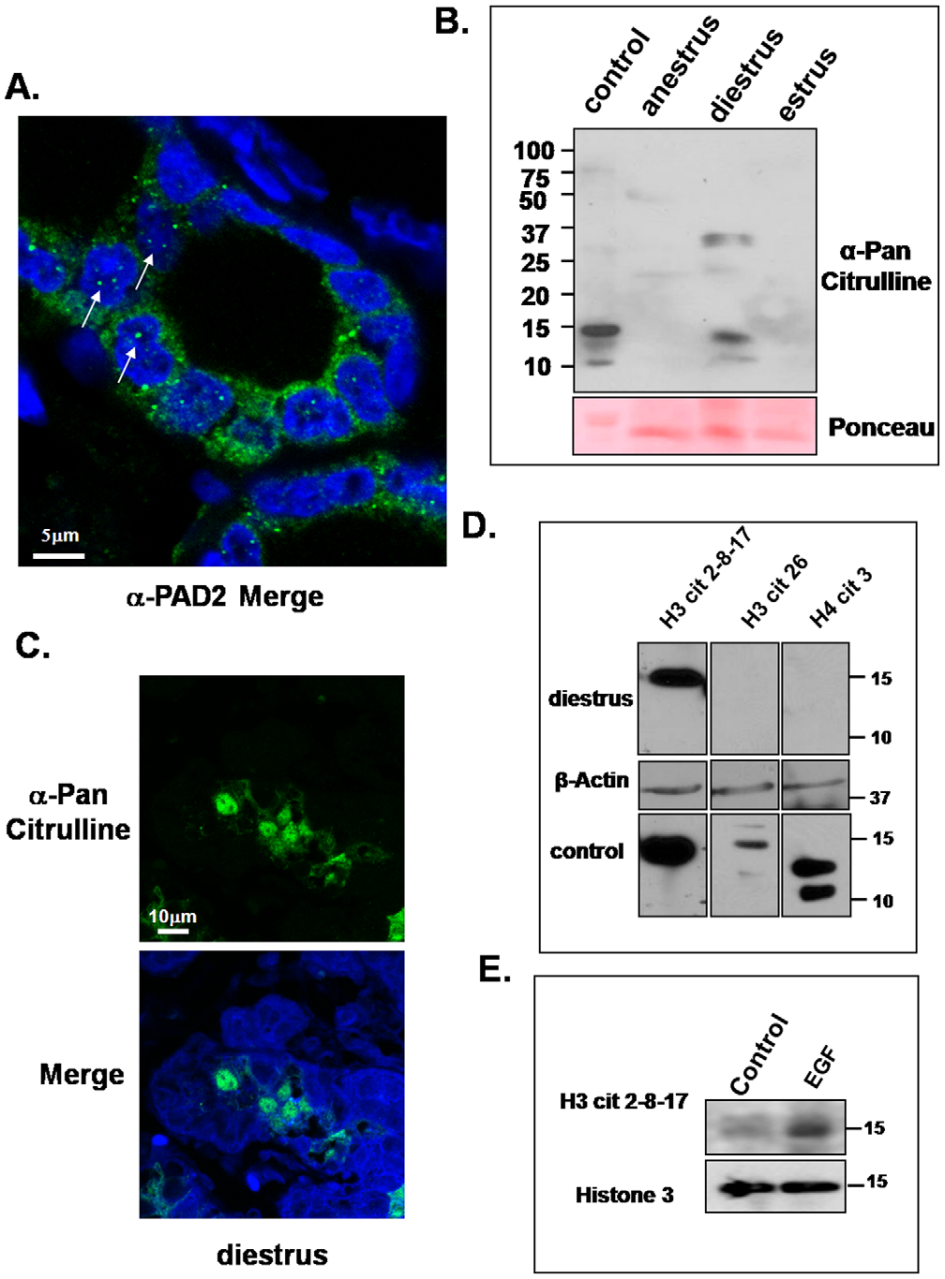
PAD2 association with histone citrullination. (A) PAD2 is detected in the nucleus and cytoplasm of mammary epithelial. PAD2 expression is shown in green and nuclear DAPI stain is in blue with white arrows denoting punctuate PAD2 staining in euchromatic nuclear regions (1000×). (B) PADs target histones from mammary gland epithelial cells for citrullination and this activity is restricted to late diestrus. Canine mammary tissue samples from anestrus, estrus/early diestrus and late diestrus were examined by SDS-PAGE and probed with an anti-pan-citrulline antibody. (C) Late diestrus mammary tissue sections were subject to a modified IF protocol using the anti-pan-citrulline antibody. The panel shows pan-citrulline staining in green and nuclear DAPI stain in blue (1000×). (D) Late diestrus canine mammary tissue samples and in vitro citrullinated CMT25 histones (as a positive control) were examined by SDS-PAGE and probed with the histone tail citrullination specific antibodies H3cit 2-8-17, H3cit 26, or H4cit 3. (E) Western blot analysis of CMT25 cells treated with EGF for 24 hours show increased histone tail citrullination using a H3 cit 2-8-17 specific antibody. Equal loading was confirmed using a mouse histone 3 antibody.

Interestingly, initial IHC and IF studies suggested that a fraction of PAD2 localized to the nucleus. To further investigate the subcellular localization of PAD2, mammary tissue sections were evaluated by IF, and high magnification images were taken. Results show that in diestrus samples, PAD2 can be visualized as punctate foci in euchromatic (i.e. nuclear regions that are stained poorly by DAPI) regions of the nucleus suggesting that PAD2 enzymatic targets may be nuclear in nature (Figure 4A, arrows). While a role for PAD2 in nuclear function has not been previously described, reports have shown that PAD2 can target histones for citrullination in vitro [12].

### PADs target histone H3 from mammary gland epithelial cells for citrullination only during diestrus and EGF can stimulate histone H3 citrullination

The expression pattern and functional role of PAD catalyzed citrullination in mammary tissue has not previously been examined. To address this issue, canine mammary tissue sections from anestrus, estrus/early diestrus, and late diestrus were assessed by western blot analysis with an antibody that recognizes citrullinated proteins (anti-pan-citrulline antibody) as previously described [21]. Pan-citrulline western blots detected three strong bands corresponding to 33, 15, and 10 kDa in diestrus canine mammary tissue samples while citrullinated proteins were not detected (or weakly detected) at other stages (Figure 4B). Based on their molecular weights, we speculate that the 33 kDa band is nucelophosmin, a known PAD substrate, and that the 10 and 15 kDa bands are likely histone H3 and H4. To further validate that the lower molecular weight proteins from the diestrus sample were histones, we citrullinated bulk histones using purified PAD2 and found that similarly sized histone bands at 10 and 15 kDa were observed (Figure 4B, control).

In order to establish where PAD-mediated citrullination was occurring within mammary tissue, sections from anestrus, estrus/early diestrus and late diestrus were subject to IF and interrogated with the pan-citrulline antibody. As expected, only the late diestrus samples showed fluorescent pan-citrulline signal. Further, we also found that this signal was primarily limited to the nucleus of a subset of epithelial cells (Figure 4C). Our finding that mammary gland epithelial cell histones appear to be a target for citrullination at diestrus raises the exciting possibility that one function of PADs in mammary tissue is to regulate gene activity via histone citrullination. To further test this hypothesis, western blots of diestrus mammary tissue samples were probed with the H3 cit 2-8-17, H3 cit 26, and H4 cit 3 site-specific anti-citrullinated histone antibodies. Purified PAD2 in vitro citrullinated CMT25 cell histones were included as a positive control. Interestingly, we found that citrullination was limited to the arginine residues at 2, 8, and 17 on the histone H3 tail suggesting that these residues are the primary targets of PAD activity in mammary epithelial cells (Figure 4D). Next we examined the ability of EGF, presumably acting via PAD2, to induce citrullination of histone H3 tails by western blot. EGF treated CMT25 cells show an increase citrullination of histone 3 tail residues 2, 8, and 17 similar to what we found in mammary tissue suggesting a role for EGF in not only PAD2 expression, but also activity (Figure 4E). Given that PAD2 activity is restricted to diestrus in vivo, the main secretory phase of the estrous cycle, our findings support the hypothesis that PAD2 may play a role in the regulation of the expression of lactation related genes in mammary epithelial cells via histone citrullination, and we are currently directly testing this hypothesis.

## Discussion

Canine mammary glands undergo dramatic molecular and morphological reorganization during the estrous cycle to convert mammary tissue to an active secretory gland. Specific stages of the estrous cycle are defined by morphologic and molecular signatures such as generalized edema and proliferation during estrus/early diestrus, production of lactation related gene products during late diestrus and apoptosis during involution towards the metestrus to anestrus crossover. These dramatic cellular transformations are clearly orchestrated, in part, by sex steroid hormones. However, the downstream targets and molecular mechanisms catalyzed by these hormones that effect cellular reorganization are poorly understood. Given that PADs are regulated by E_2_ in rodents and that citrullination of target proteins by PADs has been found to cause dramatic reorganization in both the cytoplasm and nuclear architecture, these enzymes are strong candidates for mediating hormonal changes throughout the canine estrous cycle.

IHC and IF studies of canine mammary tissue illustrate that the patterns and intensity of PAD2 expression change over the estrous cycle. For example, in estrus/early diestrus PAD2 expression is sparse, yet distinct, in cells within the alveolar end units. During diestrus, PAD2 expression is more widespread potentially indicating that, although expression is initiated during estrus, expression expands to additional epithelial cells throughout diestrus. Given this expression pattern, we hypothesize that PAD2 may possibly be involved in converting the mammary gland to an active secretory organ. Studies in rodent models have also suggested that PAD2 may be involved in a secretory function in the uterus [22].

QPCR analysis of mammary tissue samples from anestrus, estrus/early diestrus and late diestrus indicate that expression of PAD2 increases from estrus/early diestrus through late diestrus suggesting that hormonal changes during the estrus cycle may be involved in regulation of PAD2 expression. Thus, the ovarian sex steroids E_2_ and P_4_ would be appear to be the most likely candidates to regulate PAD2 expression in vivo, however our results using the CMT25 cell line indicate that E_2_ and P_4_ are likely not directly involved in regulation of PAD2 in vitro. Although the CMT cell lines are derived from canine mammary epithelial cells, their ability to respond to E_2_ and P_4_ is currently unknown and can therefore not be conclusively ruled out in contributing to PAD2 expression. In contrast, EGF can directly increase PAD2 expression in vitro. Growth factors are believed to establish auto-, para- and juxtacrine loops that are thought to augment and amplify initial steroid hormone signals. Therefore, the regulation of PAD2 expression in vivo is likely multifactoral. Interestingly, EGF receptor levels in canine mammary tissue increase from estrus through the early and mid-luteal phase thus paralleling the observed increase in PAD2 expression [23]. Additionally, we show that there is increased citrullination of CMT25 histone 3 tail arginine residues 2, 8, and 17 after EGF treatment, further supporting our hypothesis that histones are targets for PAD2. Induction of citrullination by EGF has not previously been reported and our lab is currently working to understand the up- and downstream signaling pathways that mediate this increase in PAD2 and its catalytic activity.

One unexpected result from our IHC and IF experiments is that, in addition to expected cytoplasmic and peri-nuclear localization, PAD2 is also detected in the nucleus of mammary epithelial cells. To date PAD2 expression and function in other tissues is thought to be limited to the cytoplasm. Thus, our finding suggests that PAD2 localization and function may be dependent to some degree upon the cell type in which it is expressed, and that the role and targets of PAD2 in the mammary gland may be different from those of other tissue types. Presently, studies are underway to determine the possible mechanism by which PAD2 translocates to the cell nucleus in mammary epithelial cells.

Given that no previous studies have documented PAD expression in the mammary gland nor have previous reports demonstrated that PAD-mediated citrullination occurs in mammary tissue, we believe that this study provides significant new insight into PAD biology. For example, our finding that PAD-mediated citrullination in mammary tissue appears to be restricted to epithelial cells during a distinct stage of the estrous cycle and limited to a small subset of targets (likely histones and nucleophosmin) suggests that PAD activity is likely facilitating a specific event during diestrus. Further, the observation that histone citrullination correlates with PAD2 expression in mammary epithelium at diestrus and that a fraction of PAD2 is nuclear in these cells, raises the possibility that PAD2 may play a role regulating gene activity in the mammary epithelium. Currently, we are testing whether the observed histone citrullination activity is directly regulated by PAD2 or is possibly indirectly regulated by PAD2 via crosstalk with PAD4. Subsequent findings directly demonstrating that PADs regulate gene activity in the mammary gland epithelium would likely lead to the identification of new regulatory pathways important for mammary function.

## Supporting Information

Figure S1 Immunohistochemitry in canine mammary with PAD-4. (A) As an IHC control, anestrus, estrus/early diestrus, and late diestrus mammary tissue sections were probed with equal concentration of rabbit IgG (compared to anti-PAD2) and counterstained with hematoxylin. (B) Anestrus, estrus/early diestrus, and late diestrus mammary tissue sections were are also probed with an anti-PAD4 antibody, and opposite of PAD2 staining, showed highest PAD4 levels in anestrus with lowest staining during late diestrus.
(2.96 MB TIF)

Figure S2 The PAD2 antibody is not cross reactive with other PAD family members. Mammalian expression plasmids containing the cDNA for human PADs 1, 2, 3, and 4 were transiently transfected into human embryonic kidney 293 cells. After 24 hours, cells were harvested and overexpression lysates run on a western blot probed with anti-PAD2.
(0.12 MB TIF)
