# Supplementary figures and images for: Potential Role for Peptidylarginine Deiminase 2 (PAD2) in Citrullination of Canine Mammary Epithelial Cell Histones
Source: PLoS One. 2010 Jul 26;5(7):e11768. doi: 10.1371/journal.pone.0011768 (PMC2909897; doi:10.1371/journal.pone.0011768)

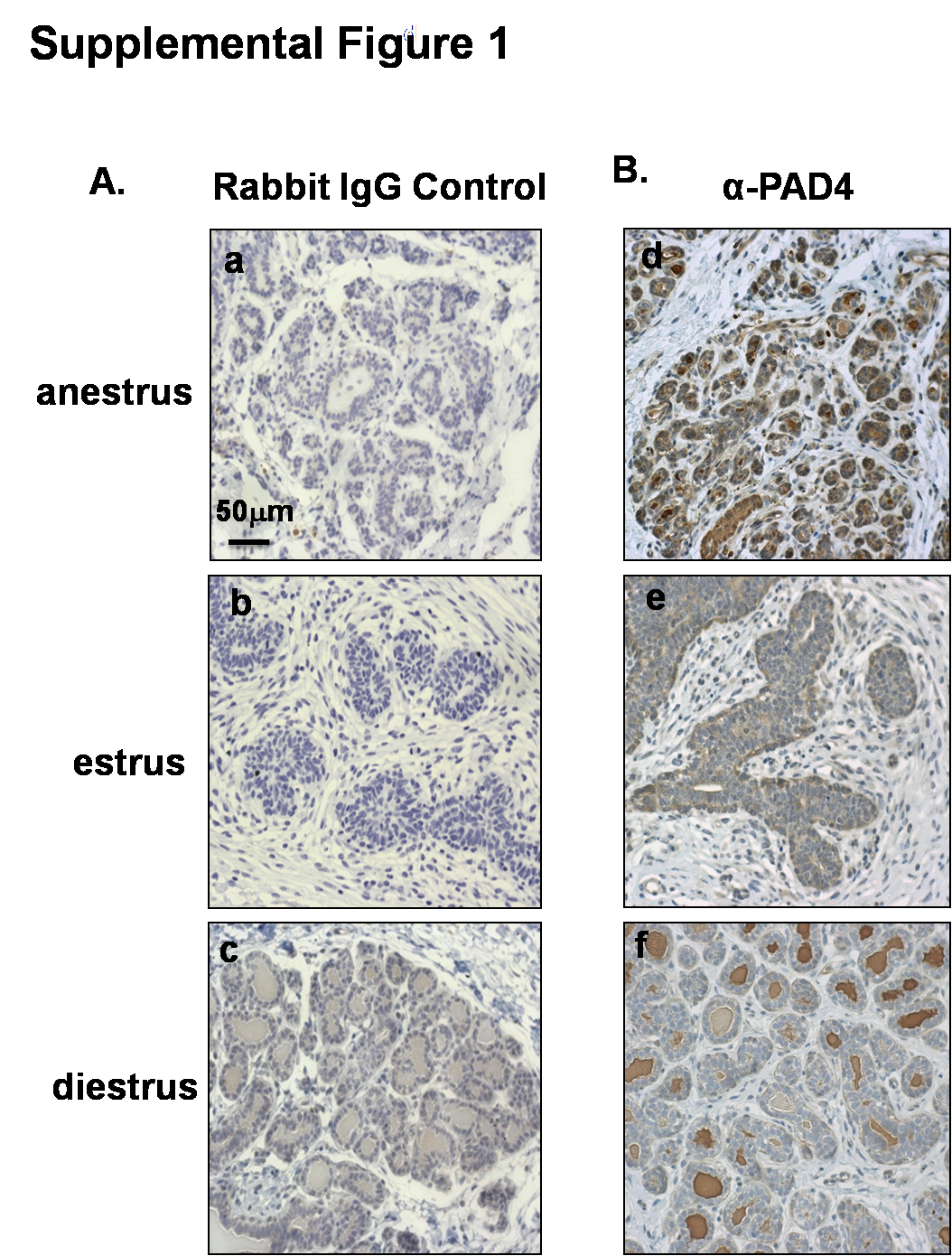

Supplement: Figure S1 — Immunohistochemitry in canine mammary with PAD-4. (A) As an IHC control, anestrus, estrus/early diestrus, and late diestrus mammary tissue sections were probed with equal concentration of rabbit IgG (compared to anti-PAD2) and counterstained with hematoxylin. (B) Anestrus, estrus/early diestrus, and late diestrus mammary tissue sections were are also probed with an anti-PAD4 antibody, and opposite of PAD2 staining, showed highest PAD4 levels in anestrus with lowest staining during late diestrus. (2.96 MB TIF) [file pone.0011768.s001.tif]

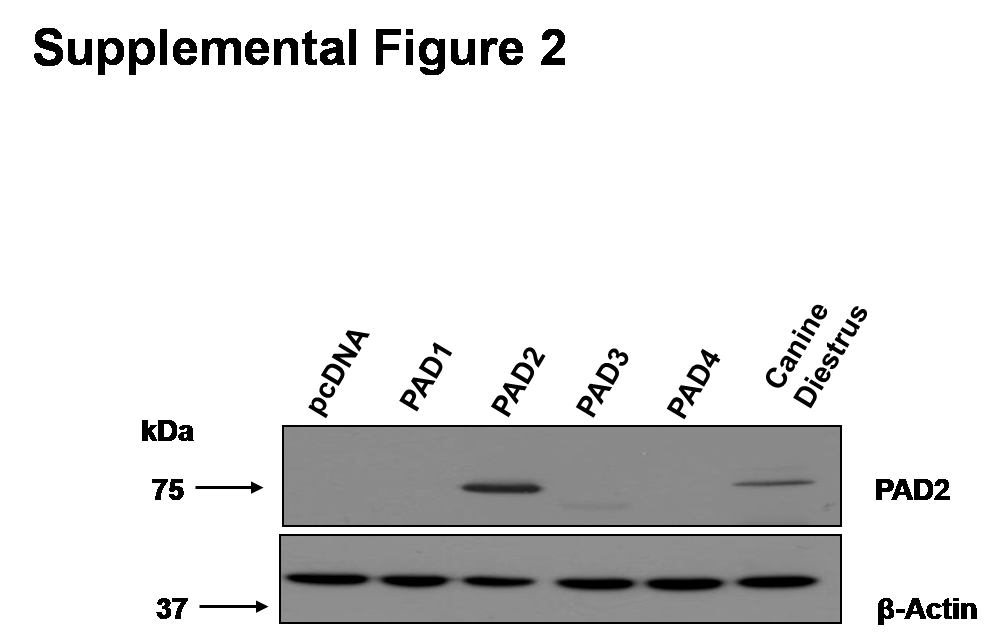

Supplement: Figure S2 — The PAD2 antibody is not cross reactive with other PAD family members. Mammalian expression plasmids containing the cDNA for human PADs 1, 2, 3, and 4 were transiently transfected into human embryonic kidney 293 cells. After 24 hours, cells were harvested and overexpression lysates run on a western blot probed with anti-PAD2. (0.12 MB TIF) [file pone.0011768.s002.tif]
